# Clinical impact of pneumothorax in patients with *Pneumocystis jirovecii* pneumonia and respiratory failure in an HIV-negative cohort

**DOI:** 10.1186/s12890-021-01812-z

**Published:** 2022-01-08

**Authors:** Ji Soo Choi, Se Hyun Kwak, Min Chul Kim, Chang Hwan Seol, Sung Ryeol Kim, Byung Hoon Park, Eun Hye Lee, Seung Hyun Yong, Ah Young Leem, Song Yee Kim, Sang Hoon Lee, Kyungsoo Chung, Eun Young Kim, Ji Ye Jung, Young Ae Kang, Moo Suk Park, Young Sam Kim, Su Hwan Lee

**Affiliations:** 1 Division of Pulmonology, Allergy and Critical Care Medicine, Department of Internal Medicine, Yongin Severance Hospital, Yonsei University College of Medicine, Yongin, Republic of Korea; 2 Division of Pulmonary and Critical Care Medicine, Department of Internal Medicine, Severance Hospital, Yonsei University College of Medicine, 50 Yonsei-ro, Seodaemun-gu, Seoul, 03722 Republic of Korea

**Keywords:** Pneumonia, Pneumocystis, Respiratory insufficiency, Pneumothorax, Prognosis, Risk factors

## Abstract

**Background:**

*Pneumocystis jirovecii* pneumonia (PCP) with acute respiratory failure can result in development of pneumothorax during treatment. This study aimed to identify the incidence and related factors of pneumothorax in patients with PCP and acute respiratory failure and to analyze their prognosis.

**Methods:**

We retrospectively reviewed the occurrence of pneumothorax, including clinical characteristics and results of other examinations, in 119 non-human immunodeficiency virus patients with PCP and respiratory failure requiring mechanical ventilator treatment in a medical intensive care unit (ICU) at a tertiary-care center between July 2016 and April 2019.

**Results:**

During follow up duration, twenty-two patients (18.5%) developed pneumothorax during ventilator treatment, with 45 (37.8%) eventually requiring a tracheostomy due to weaning failure. Cytomegalovirus co-infection (odds ratio 13.9; *p* = 0.013) was related with occurrence of pneumothorax in multivariate analysis. And development of pneumothorax was not associated with need for tracheostomy and mortality. Furthermore, analysis of survivor after 28 days in ICU, patients without pneumothorax were significantly more successful in weaning from mechanical ventilator than the patients with pneumothorax (44% vs. 13.3%, *p* = 0.037). PCP patients without pneumothorax showed successful home discharges compared to those who without pneumothorax (*p* = 0.010).

**Conclusions:**

The development of pneumothorax increased in PCP patient with cytomegalovirus co-infection, pneumothorax might have difficulty in and prolonged weaning from mechanical ventilators, which clinicians should be aware of when planning treatment for such patients.

## Background

The incidence of *Pneumocystis jirovecii* pneumonia (PCP) in patients without human immunodeficiency virus (HIV) has increased as more patients receive chemotherapy or immunosuppressive agents [1]. The disease progress in these patients is rapid, and the prognosis is worse compared to that of PCP patients with HIV [2]. Furthermore, when complicated by respiratory failure, the prognosis is poor and mortality rate is high [3].

Pneumothorax is one of the complications of PCP [4]. The prevalence of pneumothorax ranged from 13 to 61% in PCP patients with and without HIV in previous study [5]. There are several studies on the association between the occurrence of pneumothorax in PCP and prognosis in patients with HIV [6–8]. In one study of patients without HIV, development of pneumothorax was related to poor prognosis, including high acutely physiology and chronic health evaluation (APACHE) III scores, prolonged positive pressure ventilation, and intubation delay, in patients with acute respiratory failure complicating PCP [9]. And, the management of patients with PCP was more difficult when pneumothorax develops [10]. However, the relationship between pneumothorax and prognosis of PCP with acute respiratory failure in patients without HIV remains unclear [5].

The aim of this study was to analyze the incidence and related risk factors of pneumothorax in non-HIV patients with PCP and acute respiratory failure, and to further identify the clinical impact in those patients.

## Methods

### Study population

In this study, we retrospectively investigated the medical records of 1,210 patients who were admitted to a medical intensive care unit at a tertiary care university hospital in South Korea between July 2016 and April 2019. Patients with PCP without HIV who needed mechanical ventilation due to respiratory failure in ICU care were included. Three criteria were applied for the diagnosis of PCP: (1) immunocompromised status from chemotherapy, immunosuppressant usage, or long term use of steroid with clinical symptoms of pneumonia, such as cough, sputum, fever, and dyspnea; (2) *P. jirovecii* DNA must be confirmed using polymerase chain reaction (PCR) assays from patient sputum samples, endotracheal aspirates, or bronchoalveolar lavage fluids; and (3) radiologic finding from chest computed tomography (CT) representing typical patterns of PCP, including bilateral interstitial opacities, ground glass opacities, or septal thickening must be evident. *P. jirovecii* PCR-positive patients who did not receive treatment for PCP were excluded due to possible false positivity.

### Data collection

Patient baseline characteristics, laboratory findings, and information on disease severity, such as APACHE II score, Sequential Organ Failure Assessment (SOFA) score, and Simplified Acute Physiology Score (SAPS) II, were collected from data obtained within 24 h of ICU admission. We reviewed the mechanical ventilator (MV) parameters, including respiratory rate, tidal volume (mL/predicted body weight), peak inspiratory pressure, positive expiratory end pressure (PEEP), dynamic driving pressure (the difference between peak inspiratory pressure and PEEP) within 24 h after MV initiation. We also reviewed peak inspiratory pressure three days after- and maximal peak pressure within a week after MV initiation. Concomitant Cytomegalovirus (CMV) antigenemia was decided through a quantitative PCR test. The cutoff value for clinically meaningful positive CMV PCR result was higher or equal 1500 copies/mL [11].

Occurrence of pneumothorax was evaluated via radiologic manifestations with chest radiography and CT. We investigated the prognosis of patients, including successful MV weaning outcomes, ICU length of stay, 28-day mortality and in-hospital mortality. Approval for this study was provided by the institutional review board of Yongin Severance Hospital (IRB 9-2021-0045). The need for informed consent was waived due to the retrospective nature of this study. This study was conducted in accordance with the tenets set by the Declaration of Helsinki.

### Management of PCP

All study patients were administered intravenous or oral trimethoprim (15–20 mg/kg per day) and sulfamethoxazole (75–100 mg/kg per day) as first-line treatment for PCP. The duration of planned treatment was three weeks. We changed to second-line treatment using primaquine (15–30 mg/day) and clindamycin (1800 mg/day) or pentamidine (4 mg/kg per day) in patients who did not present clinical improvement. Twenty-one (17.6%) patients were treated with second-line medication due to no clinical improvement or side effects of the first-line medication. In addition, all patients were administered adjuvant corticosteroid (40 mg prednisolone twice daily for five days, followed by 40 mg prednisolone twice daily for five days, after which 20 mg prednisolone twice daily for 11 days was given). Patients requiring MV treatment for more than two weeks due to MV weaning failure underwent tracheostomy.

### Statistical analysis

Categorical variables were presented as frequencies and percentages with comparisons done using a chi-square test. Continuous variables were presented as means and standard deviations if the distribution was normal, and as interquartile ranges (IQRs) if the distribution was not normal. For comparisons of continuous variables, a Student’s t-test and Mann–Whitney U test were used. A multivariable logistic regression analysis was performed with pre-specified covariates. Odds ratios (ORs) and 95% confidence intervals (CIs) were also calculated. A *p*-value < 0.05 was considered significant for all analysis. All data were analyzed statistically using IBM SPSS version 25.0 software (IBM Corp., Armonk, NY, USA).

## Results

### Study flow

**Fig. 1:**
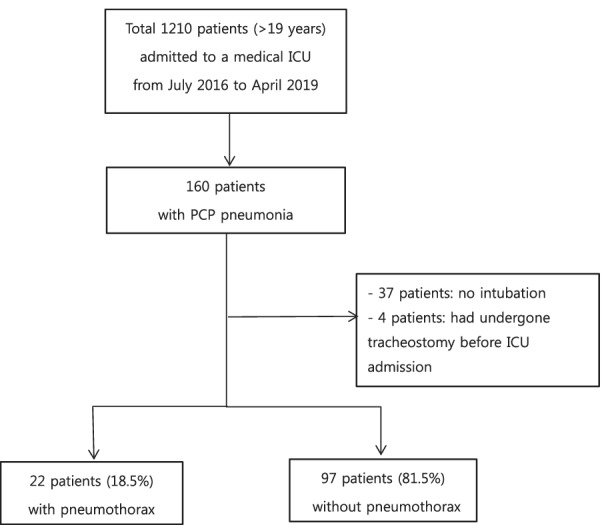
Flow diagram representing patient enrollment. ICU, intensive care unit, PCP, *Pneumocystis jirovecci* pneumonia

A total of 160 patients with clinically confirmed PCP and on treatment for it were enrolled. Forty one patients who had respiratory failure, but had not received MV treatment and had undergone tracheostomy before ICU admission were excluded; therefore, 119 patients were finally included in our study (Fig. 1).

### Baseline characteristics of the study participants

The baseline characteristics of study patients are summarized in Table 1. Among the 119 patients reviewed, 76 (63.9%) were male and 43 (36.1%) were female. Median age was 65.0 (IQR, 56.0–72.0) years. Median APACHE II score was 26.5 (IQR, 20.0–32.0). Thirty-four patients (28.6%) were smokers, and 31 (26.1%) had underlying chronic lung diseases, including airway disease (n = 9, 7.3%) and interstitial lung disease (n = 22, 18.5%). The main cause of immunodeficiency was a solid cancer (n = 37, 31.1%), followed by immunosuppressive agent use (n = 27, 22.7%), and hematologic malignancy (n = 22, 18.5%). Forty-one patients (34.5%) needed continuous renal replacement therapy (CRRT) because of acute kidney injury. Concomitant CMV antigenemia was noted in 75 patients (63.0%).

**Table 1:** Baseline characteristics of patients with *Pneumocystis jirovecci* pneumonia requiring mechanical ventilation in intensive care unit

Patient characteristics	N = 119
Median age, years	65.0 (56.0–72.0)
Sex (male)	76 (63.9)
Height, cm	163.0 (156.0–170.0)
BMI, kg/m^2^	22.3 (20.1–25.0)
Ever smoker	34 (28.6)
Charlson Comorbidity Index	3 (2–4)
**Comorbidity disease**	
Congestive heart failure	11 (9.2)
Coronary arterial disease	21 (17.6)
Chronic lung disease—airway	9 (7.6)
Chronic lung disease—ILD	22 (18.5)
Chronic kidney disease	31 (26.1)
Chronic liver disease	9 (7.6)
Cerebrovascular disease	4 (3.4)
Solid cancer	37 (31.1)
Hematologic malignancy	22 (18.5)
Immunosuppressive agent use	27 (22.7)
CRRT due to AKI	41 (34.5)
APACHE II score	26.5 (20.0–32.0)
SOFA score	8 (6–11)
SAPS II	37.0 (29.0–18.8)
Cytomegalovirus antigenemia	75 (63.0)
Clinical parameters	
WBC, × 10^3^/μL	10.2 (5.9–15.3)
Platelet, × 10^3^/μL	132.5 (60.8–246.0)
BUN, mg/dL	29.5 (19.5–44.6)
Creatinine, mg/dL	1.07 (0.62–1.90)
Albumin, g/dL	2.5 (2.2–2.7)
Lactate, mmol/L	2.0 (1.5–4.4)
C-reactive protein, mg/L	117.7 (68.4–189.0)
Procalcitonin, ng/mL	1.4 (0.3–4.3)
Pneumothorax	22 (18.5)
Tracheostomy	45 (37.8)
28-day mortality	45 (37.8)

Data are presented by numbers (%) or median (IQR) unless indicated otherwise

*IQR* interquartile range, *BMI* body mass index, *ILD* interstitial lung disease, *CRRT* continuous renal replacement therapy, *AKI* acute kidney injury, *APACHE II* acute physiology and chronic health evaluation II, *SOFA* sequential organ failure assessment, *SAPS II* simplified acute physiology score II, *WBC* white blood cell, *BUN* blood urea nitrogen

### Factors and outcomes associated with occurrence of pneumothorax

In total, 22 patients (18.5%) developed pneumothorax during MV treatment, with the median time from initiating ventilator management to development of pneumothorax being 8.0 days (IQR, 1.5–16.0). Table 2 shows comparisons of associated factors and outcomes, according to occurrence of pneumothorax. On univariate analysis, low body mass index (BMI), underlying airway disease, renal failure requiring CRRT, low procalcitonin level, low SOFA score, and CMV antigenemia were associated with the development of pneumothorax in patients with PCP and respiratory failure. However, there was no significant difference in MV parameters within 24 after MV initiation, including tidal volume, peak pressure, and dynamic driving pressure. Peak pressure at three days after MV initiation and maximal peak pressure seven days after MV initiation did not show statistical significance between those with and without pneumothorax. Furthermore, there was no significant difference in proportion of patients who had undergone tracheostomy and mortality rate, according to presence or absence of pneumothorax.

**Table 2:** Comparison of characteristics according to occurrence of pneumothorax

	No pneumothorax	Pneumothorax	*p*-value	Multivariate analysis	
	N = 97 (81.5%)	N = 22 (18.5%)		OR (95% CI)	*p-* value
Median age, years	65.0 (55.0–71.5)	64.5 (58.0–72.0)	0.760	1.0 (0.958–1.047)	0.949
Sex (male)	62 (63.9)	14 (63.6)	0.980	1.3 (0.440–3.895)	0.628
Height, cm	163.0 (156.0–169.5)	165.0 (156.8–170.8)	0.415		
BMI, kg/m^2^	22.7 (20.5–25.7)	20.8 (19.5–23.0)	0.012	1.2 (1.011–1.356)	0.035
Ever smoker	28 (28.9)	6 (27.3)	0.881		
Charlson Comorbidity Index	3 (2–4)	2 (2–3.5)	0.447		
Underlying disease					
Congestive heart failure	8 (8.2)	3 (13.6)	0.431		
Chronic lung disease—airway	5 (5.2)	4 (18.2)	0.037	0.2 (0.050–1.160)	0.076
Chronic lung disease—ILD	17 (17.5)	5 (22.7)	0.553		
Chronic kidney disease	27 (27.8)	4 (18.2)	0.352		
Chronic liver disease	8 (8.2)	1 (4.5)	1.0		
Solid cancer	31 (32.0)	6 (27.3)	0.801		
Hematologic malignancy	20 (20.6)	2 (9.1)	0.360		
History of lung operation	8 (8.2)	5 (22.7)	0.063		
History of pneumothorax	0 (0)	1 (4.5)	0.185		
CRRT due to AKI	38 (39.2)	3 (13.6)	0.023		
Clinical parameters					
WBC, × 10^3^/μL	9.9 (5.7–14.8)	11.5 (6.1–22.4)	0.162		
Lactate, mmol/L	2.2 (1.5–5.1)	1.8 (1.4–2.1)	0.130		
C-reactive protein, mg/L	129.2 (69.9–208.2)	100.5 (10.1–160.2)	0.108		
Procalcitonin, ng/mL	1.5 (0.5–5.2)	0.3 (0.2–0.5)	0.008		
PaO_2_/FiO_2_ ratio at admission day	83.6 (66.7–111.0)	79.4 (61.6–101.1)	0.372		
PaO_2_/FiO_2_ ratio at 3-day after admission	38.2 (31.0–43.5)	39.2 (31.7–49.3)	0.446		
PaO2 at admission day	87.7 (70.1–117.4)	94.1 (80.7–108.9)	0.433		
PaO2 at 3-day after admission	84.6 (71.2–100.5)	74.4 (63.5–85.5)	0.063		
A-a gradient at admission day	263.8 (165.9–458.5)	338.0 (262.2–427.7)	0.556		
A-a gradient at 3-day after admission	197.6 (127.6–318.1)	267.1 (148.0–350.5)	0.324		
APACHE II score	27 (21–32)	21 (18–32)	0.129		
SOFA score	8.0 (6.0–12.0)	7.0 (4.5–8.0)	0.003		
SAPSII	37.0 (30.0–51.0)	35.0 (24.5–45.0)	0.134		
Bacterial respiratory infection	12 (12.4)	3 (13.6)	1.000		
Fungal respiratory infection	7 (7.2)	0 (0)	0.346		
Cytomegalovirus antigenemia	54 (55.7)	21 (95.5)	< 0.001	13.9 (1.737–111.072)	0.013
Tracheostomy	34 (35.1)	11 (50)	0.192		
28-day mortality	38 (39.2)	7 (31.8)	0.521		
In-hospital mortality	68 (70.1)	17 (77.3)	0.502		
Mechanical ventilator parameters					
Respiratory rate, /min	20.0 (16.0– 24.0)	20.0 (18.0–26.0)	0.379		
Tidal volume at admission day, mL/kg	6.7 (6.1–7.6)	6.8 (6.2–7.5)	0.405		
Peak pressure at admission day, cmH_2_O	25.0 (22.0–30.0)	26.0 (22.8–32.3)	0.420		
Peak pressure at 3-day after admission, cmH_2_O	26.0 (20.3–30.0)	26.0 (21.0–34.5)	0.357		
Maximal peak pressure, cmH_2_O	29.0 (25.0–34.0)	31.5 (26.0–38.5)	0.205		
Positive expiratory end pressure, cmH_2_O	7.0 (5.0–10.0)	5.5 (5.0–8.0)	0.069		
Dynamic driving pressure, cmH_2_O	19.0 (15.0–26.8)	17.0 (14.0–22.0)	0.166		

Data were presented by numbers (%) or median (IQR) unless otherwise indicated

*IQR* interquartile range, *BMI* body mass index, *ILD* interstitial lung disease, *CRRT* continuous renal replacement therapy, *AKI* acute kidney injury, *PaO2* partial pressure of arterial oxygen, *FiO2* fraction of inspired O2 concentration, *APACHE II* Acute Physiology and Chronic Health Evaluation II, *SOFA* Sequential Organ Failure Assessment, *SAPS II* simplified acute physiology score II, *WBC* white blood cell; BUN, blood urea nitrogen

Age, sex, and variables related to pulmonary involvement, including BMI, underlying airway disease, and CMV antigenemia, with a *p*-value < 0.05 in the univariate analysis were used in the multivariate analysis. Low BMI (OR, 1.2; 95% 67 CI, 1.011–1.356) and CMV antigenemia (OR 13.9; 95% CI, 1.737–111.072) remained significant risk factors in the multivariate analysis.

### The 28-day mortality in ICU and prognosis of PCP with respiratory failure

The 28-day mortality rate in ICU was 62.2% (n = 45). The in-hospital mortality rate was 71.4% (n = 85). Patients with renal failure requiring CRRT were at significantly higher mortality(*p* = 0.001). High disease severity, including high APACHE II score (*p* = 0.045), SOFA score (*p* < 0.001), and SAPS II (*p* = 0.015), was related to poor outcomes in patients with PCP. Regarding MV parameters, the peak pressure three days after admission (*p* = 0.003) and maximal peak pressure (*p* = 0.009) were higher in 28-day mortality group than in 28-day survivor groups. In the multivariate analysis, renal failure requiring CRRT (OR, 6.645; 95% CI, 1.967–22.446) was significantly associated with poor prognosis in patients with PCP and respiratory failure. (Table 3).

**Table 3:** Comparison of the characteristics according to 28-day mortality

	The 28-day survivor	The 28-day non-survivor	*p*-value	Multivariate analysis	
	N = 74 (62.2%)	N = 45 (37.8%)		OR (95% CI)	*p*-value
Median age	60.9 (57–72)	64 (55.5–71.5)	0.587		
Sex (male)	48 (64.9)	28 (62.2)	0.771	0.585(0.193–1.779)	0.345
Height, cm	164.0 (156.0– 170.0)	161.8 (156.0– 168)	0.616		
BMI, kg/m^2^	22.4 (20.4–25.4)	22.1 (19.8–24.1)	0.511		
Ever smoker	21 (28.4)	13 (28.9)	0.952		
Charlson Comorbidity Index	3 (2– 4)	3 (2– 5.5)	0.281		
Comorbidity disease					
Congestive heart failure	4 (5.4)	7 (15.6)	0.100		
Chronic lung disease—airway	4 (5.4)	5 (11.1)	0.296		
Chronic lung disease—ILD	15 (20.3)	7 (15.6)	0.521		
Chronic kidney disease	17 (23)	14 (31.1)	0.327		
Chronic liver disease	6 (8.1)	3 (6.7)	1.0		
Solid cancer	21 (28.4)	16 (35.6)	0.412		
Hematologic malignancy	12 (16.2)	10 (22.2)	0.413		
CRRT due to AKI	17 (23)	24 (53.3)	0.001	6.645(1.967–22.446	0.002
Clinical parameters					
WBC, × 10^3^/μL	10.8 (6.8–15.7)	8.5 (4.0–14.6)	0.070		
Hct, %	28.0 (24.9–32.6)	25.6 (22.9–29.6)	0.045		
Platelet, × 10^3^/μL	153.5 (79.5–263)	79 (28–179)	0.001	1.001(0.996–1.007)	0.658
BUN, mg/dL	27.2 (18.6–39.4)	35.2 (22.9–52.0)	0.069		
Creatinine, mg/dL	1.1 (0.6–1.8)	1.1 (0.6–2)	0.810		
Albumin, g/dL	2.5 (2.3–2.7)	2.3 (2.0–2.6)	0.082		
Total bilirubin, mg/dL	0.5 (0.3–1.0)	0.9 (0.4–3.4)	0.019	0.927(0.803–1.071)	0.304
Sodium, mmol/L	135 (132–138)	139.5 (132.8– 141.8)	0.248		
Potassium, mmol/L	4.4 (3.6–5.0)	4.4 (3.8–5.0)	0.855		
Lactate, mmol/L	1.8 (1.2–4.3)	3.1 (1.5–13.1)	0.054		
Procalcitonin, ng/mL	1.4 (0.3–5.1)	1.4 (0.3–10.9)	0.556		
C-reactive protein, mg/L	113.6 (50.4–182.1)	137.9 (71.7–227.8)	0.253		
PaO_2_/FiO_2_ ratio at admission day	79.2 (64.9–107.8)	86.4 (65.3–108.3)	0.569		
PaO_2_/FiO_2_ ratio at 3-day after admission	189.0 (133.3–278.9)	134.6 (88.0–212.0)	0.006	1.004(0.995–1.014)	0.371
PaO2 at admission day	93.3 (74.7–121.3)	81.9 (69.4–107.9)	0.268		
PaO2 at 3-day after admission	82.3 (73.9–107.0)	76.2 (64.9–95.6)	0.092		
A-a gradient at admission day	291.7 (165.1–426.2)	317.4 (190.7–488.6)	0.391		
A-a gradient at 3-day after admission	162.7 (117.9–297.5)	309.7 (152.2–404.3)	0.005	1.000(0.993–1.007)	0.952
APACHE II score	25 (19.0–30.0)	28.5 (22.5–37.0)	0.045	0.998(0.929–1.073)	0.959
SOFA score	7 (6–9)	10 (8–13)	< 0.001	0.871(0.696–1.090)	0.226
SAPS II	35.0 (27–45.5)	44 (33–54)	0.015		
Pneumothorax	15 (20.3%)	7 (15.6%)	0.521		
Bacterial respiratory infection	9 (12.2)	6 (13.3)	0.852		
Fungal respiratory infection	3 (4.1)	4 (8.9)	0.424		
Cytomegalovirus antigenemia	51 (68.9)	24 (53.3)	0.088		
Mechanical ventilator parameters					
Respiratory rate, /min	20 (16.0–25.3)	20 (18.0–24.0)	0.535		
Tidal volume at admission day, mL/kg	380.0 (320.0–400.0)	350 (300.0–380.0)	0.831		
Peak pressure at admission day, cmH_2_O	27.0 (20.8–32.0)	26.0 (23.0–33.0)	0.988		
Peak pressure at 3-day after admission, cmH_2_O	25.0 (19.0–31.3)	31.0 (27.0–41.0)	0.003	0.998(0.861–1.158)	0.983
Maximal peak pressure, cmH_2_O	30.0 (24.5–35.5)	36.0 (29.0–42.0)	0.009	0.911(0.786–1.056)	0.217
Positive expiratory end pressure, cmH_2_O	5.0 (5.0–8.0)	7.0 (5.0–10.0)	0.328		
Dynamic driving pressure, cmH_2_O	18.0 (14.8–22.3)	17.0 (14.0–22.0)	0.777		

Data are presented as numbers (%) or median (IQR) unless indicated otherwise

*IQR* interquartile range, *BMI* body mass index, *ILD* interstitial lung disease, *CRRT* continuous renal replacement therapy, *AKI* acute kidney injury, *APACHE II* Acute Physiology and Chronic Health Evaluation II, *SOFA* Sequential Organ Failure Assessment, *SAPS II* simplified acute physiology score II, *WBC* white blood cell, *BUN* blood urea nitrogen

### Clinical impact of pneumothorax in surviving PCP patients.

Of 119 patients, 45 (37.8%) underwent tracheostomy due to weaning failure. The need for tracheostomy for long term MV management did not significantly differ between patients with- and without pneumothorax (Table 2).

We further investigated on the 74 study patients who survived more than 28 days in ICU. Among them, 15 (20.3%) developed pneumothorax. There was no significant difference in tracheostomy rates between those with and without pneumothorax (Table 4). However, patients without pneumothorax were significantly more successful in weaning from MV than were patients with pneumothorax (44% vs. 13.3%, *p* = 0.037). The ICU length of stay was also longer in patients with pneumothorax, although there was no statistically significant difference (*p* = 0.068). Patients with PCP and pneumothorax were significantly associated with a poor prognosis, with a mortality of 33.3% (*p* = 0.048), and only two patients (13.3%) were eventually discharged home, compared to 25 (51.0%) successful home discharges among PCP patients without pneumothorax (*p* = 0.010, Table 4).

**Table 4:** Clinical prognosis in patients with 28-day survival (n = 74)

	No pneumothorax	Pneumothorax	*p*-value
	N = 59 (79.7)	N = 15 (20.3)	
Tracheostomy	29 (49.2)	9 (60)	0.453
Ventilator weaning	26 (44)	2 (13.3)	0.037
ICU length of stay	18 (12–40)	32 (24–58)	0.068
Final survivors	29 (49.2)	5 (33.3)	0.048
Discharge to home	25 (51.0)	2 (13.3)	0.010

Data are presented as numbers (%) or median (IQR) unless indicated otherwise

*ICU* intensive care unit

## Discussion

This study described the incidence and related factors of pneumothorax in patients with PCP and their prognosis. In addition, we analyzed survival outcomes in patients with PCP and acute respiratory failure. The development of pneumothorax in PCP was not associated with increased 28-day mortality; however, patients with pneumothorax had difficulty and prolonged MV weaning. Among several factors, CMV co-infection was associated with the development of pneumothorax.

There are several complications associated with PCP [12], and pneumothorax occurs with prevalence of 5–20% [13]. The pathogenesis of pneumothorax in PCP was unclear, but had suggested to the tissue destruction from direct tissue toxicity by the pathogens, over-distention of bronchioles, and prolonged existence of macrophage with increased elastase and other enzymes [14]. As a results, cyst and bullae were formed, and parenchymal lung was vulnerable status to development of pneumothorax [6]. Pneumothorax is also a common complication during ventilator treatment, with a reported incidence of 4–15% [15–17]. Patients with acute respiratory distress syndrome are more vulnerable to the occurrence of pneumothorax, with further higher risks in patients with underlying lung disease, than those who do not have this syndrome [18]. In our study population of patients with acute respiratory failure requiring MV care, which is a risk factor for pneumothorax [19], the incidence of pneumothorax was 18.5% (22/119), which is high. Furthermore, underlying lung diseases, including airway disease and interstitial lung disease, showed relation to increased occurrence of pneumothorax in the univariate analysis, in accordance with a previous report [20].

We found that MV parameters were not related to the development of pneumothorax. Some studies showed that ventilator parameters, such as peak airway pressure, tidal volume, and PEEP, had no correlation with increased risk of pneumothorax [21–23], although earlier studies had reported relevance [24]. Miller et al. reported that protective lung strategies had an effect on decreasing barotrauma [25]. We had managed patients with tidal volumes of 6–7 mg/kg and peak pressures lower than 35 cmH_2_O, according to the lung protective strategy [26]. We showed that respiratory mechanics did not significantly affect the development of pneumothorax in patients with PCP when this lung protective strategy was applied. Furthermore, in one study, Boussarsar et al. said that barotrauma during MV care was more strongly associated with underlying lung conditions and compliance than with MV parameters [27].

In immunocompromised patients, PCP commonly presents with co-infection of other pathogens, especially CMV [28]. The reports on effect and outcome of CMV co-infection with PCP are controversial [29, 30], although there are several researches stating that concurrent infection of CMV is related to increased mortality and poor prognosis [31, 32]. Our study showed that CMV antigenemia was not associated with a high 28-day mortality rate; however, patients with CMV antigenemia were significantly associated with increased occurrence of pneumothorax. In our study populations, the effect of CMV reactivation on PCP prognosis is meaningful because the seropositivity of CMV in Koreans was reported as high as 94.1% [33]. Cook et al. suggested that CMV reactivation could cause abnormal cytokine/chemokine expression, resulting in pulmonary fibrosis in an animal model [34]. In one prior research on histopathological findings in 12 deceased patients with PCP, three had evidence of CMV co-infection, and two of them presenting with pulmonary fibrosis [35]. Furthermore, structural changes in lung parenchyma, including fibrosis, is one of proposed mechanisms of pneumothorax and increases vulnerability to the occurrence of pneumothorax [36].

Occurrence of PCP in patients without HIV has poorer progress and higher mortality than that in patients with HIV [37]. In our study, the in-hospital mortality rate was high at 71.4%, will only 28 patients (23.5%) achieving MV weaning. Pneumothorax in patients with PCP with- or without HIV was difficult to treat and had a worse prognosis than pneumothorax from other etiologies [9, 10, 19]. However, in our study, the development of pneumothorax was not associated with increased 28-day mortality. In a previous study, acute respiratory failure requiring invasive MV was found to be a risk factor for increased mortality; therefore, the effect of pneumothorax was likely to be lessened because all patients enrolled our study already had respiratory failure [37]. However, we found that development of pneumothorax in patients with PCP who required invasive MV procedures made weaning difficult.

Our study had several limitations. First, this study was retrospectively conducted at a single center. However, we enrolled a large number of patients with PCP and respiratory failure without HIV requiring invasive MV procedures. Furthermore, since all enrolled patients were applied MV, the bias of applying MV could be reduced. Second, we did not acquire microbiological findings from patient samples for diagnosis of PCP but only diagnosed via *P. jirovecii* PCR assays. However, the sensitivity and specificity of this assay for detecting *P. jirovecii* were comparable to those of microscopic staining [38]. Furthermore, we included only patients who were treated for PCP with typical symptoms of pneumonia and characteristic radiological findings. Third, all patients did not perform the chest CT and followed up chest CT. So, we did not identify the cystic changes of lung parenchyme that could affect the occurrence of pneumothorax in PCP patients. Fourth, the duration of air-leak that could affect the prognosis of PCP patients with pneumothorax and several parameter of mechanical ventilator including plateau pressure were not evaluate because our analysis was retrospective. Finally, in patients discharged to long-term outpatient care after survival, it was not possible to investigate final success of MV weaning.

Nevertheless, our study also had some strengths. We analyzed a large number of patients with PCP and acute respiratory failure, and found related factor of pneumothorax. ICU clinicians might predict that patients with PCP and CMV antigenemia have increased risk of pneumothorax. Additionally, development of pneumothorax in patients with PCP could be a predictive factor of delayed MV weaning and poor final outcome. These results could be helpful to the real clinical field ICU clinician. However, further prospective studies are needed to validate our findings.

## Conclusion

The results of this study suggest that patients with PCP who develop pneumothorax might have difficulty and delayed weaning from MV. Concomitant CMV antigenemia could be a predictive factor for pneumothorax occurrence. Therefore, clinicians need to closely observe the occurrence of pneumothorax in patient with PCP and CMV antigenemia and should anticipate that MV weaning may be difficult in such patients.

## Data Availability

The datasets generated during and/or analysed during the current study are available from the corresponding author on reasonable request.

## Abbreviations

PCP: Pneumocystis jirovecii pneumonia
ICU: Intensive care unit
HIV: Human immunodeficiency virus
APACHE: Acutely physiology and chronic health evaluation
PCR: Polymerase chain reaction
CT: Computed tomography
SOFA: Sequential Organ Failure Assessment
SAPS: Simplified Acute Physiology Score
MV: Mechanical ventilator
PEEP: Positive expiratory end pressure
IQRs: Interquartile ranges
ORs: Odds ratios
CIs: Confidence intervals
CRRT: Continuous renal replacement therapy
CMV: Cytomegalovirus
BMI: Body mass index
